# Paradoxical activation of the sodium chloride cotransporter (NCC) without hypertension in kidney deficient in a regulatory subunit of Na,K‐ATPase, FXYD2

**DOI:** 10.14814/phy2.12226

**Published:** 2014-12-03

**Authors:** Elena Arystarkhova, Donna L. Ralph, Yi Bessie Liu, Richard Bouley, Alicia A. McDonough, Kathleen J. Sweadner

**Affiliations:** 1 Laboratory of Membrane Biology, Massachusetts General Hospital Boston, Massachusetts; 2 Department of Cell and Neurobiology, Keck School of Medicine, University of Southern California Los Angeles, California; 3 MGH Center for Systems Biology, Program in Membrane Biology, Massachusetts General Hospital Boston, Massachusetts

**Keywords:** Apical Na^+^ transporters, blood pressure, distal tubule, phosphorylation

## Abstract

Na,K‐ATPase generates the driving force for sodium reabsorption in the kidney. Na,K‐ATPase functional properties are regulated by small proteins belonging to the FXYD family. In kidney FXYD2 is the most abundant: it is an inhibitory subunit expressed in almost every nephron segment. Its absence should increase sodium pump activity and promote Na^+^ retention, however, no obvious renal phenotype was detected in mice with global deletion of FXYD2 (Arystarkhova et al. 2013). Here, increased total cortical Na,K‐ATPase activity was documented in the *Fxyd2*^−/−^ mouse, without increased *α*1*β*1 subunit expression. We tested the hypothesis that adaptations occur in distal convoluted tubule (DCT), a major site of sodium adjustments. Na,K‐ATPase immunoreactivity in DCT was unchanged, and there was no DCT hypoplasia. There was a marked activation of thiazide‐sensitive sodium chloride cotransporter (NCC; Slc12a3) in DCT, predicted to increase Na^+^ reabsorption in this segment. Specifically, NCC total increased 30% and NCC phosphorylated at T53 and S71, associated with activation, increased 4‐6 fold. The phosphorylation of the closely related thick ascending limb (TAL) apical NKCC2 (Slc12a1) increased at least twofold. Abundance of the total and cleaved (activated) forms of ENaC *α*‐subunit was not different between genotypes. Nonetheless, no elevation of blood pressure was evident despite the fact that NCC and NKCC2 are in states permissive for Na^+^ retention. Activation of NCC and NKCC2 may reflect an intracellular linkage to elevated Na,K‐ATPase activity or a compensatory response to Na^+^ loss proximal to the TAL and DCT.

## Abstract (precis)

We discovered a substantial activation of renal NCC cotransporter in mice genetically depleted for the regulatory inhibitory subunit of Na,K‐ATPase, FXYD2. Surprisingly, no significant changes in urine output as well as elevation of blood pressure were detected suggesting compensatory adaptation elsewhere in nephron

## Introduction

FXYD proteins play essential roles in modulation of Na,K‐ATPase activity. The seven members of the gene family (Sweadner and Rael 2000) exhibit tissue‐ and cell‐specific distribution, and when associated with the Na,K‐ATPase they differentially modulate kinetic properties of the pump either by changing affinity for the substrates or affecting the *V*_max_ (Geering 2006). In kidney, four different FXYDs are expressed in a segment‐specific manner (Wetzel and Sweadner 2001; Capurro et al. 1996; Lubarski et al. 2005; Wetzel and Sweadner 2003), with FXYD2 being the most abundant. It has two splice variants, FXYD2a and FXYD2b, which differ only in the first exon coding for the extracellular N‐terminus of the molecule (Arystarkhova et al. 2002b; Küster et al. 2000). In rodents, only FXYD2a is found in proximal convoluted tubules (PT), while only FXYD2b is expressed in DCT and connecting tubules (CNT) (Arystarkhova et al. 2002b; Pu et al. 2001). The splice variants are coexpressed in medullary thick ascending limb. Both splice variants reduce Na^+^ affinity when expressed in stable transfectants and assayed in vitro (Arystarkhova et al. 1999, 2002a; Therien et al. 1999; Pu et al. 2002). Assays in kidney membranes from *Fxyd2*^−/−^ global knockout mice (either from outer medulla or whole kidney) confirmed that FXYD2 reduces the Na^+^ affinity of Na,K‐ATPase (Jones et al. 2005). Induction of FXYD2a by hypertonicity markedly reduced *V*_max_ in a renal cell line (Wetzel et al. 2004). Here, we demonstrated a corresponding increase in Na,K‐ATPase *V*_max_ in the knockout mouse.

The absence of FXYD2 could potentially enhance renal Na^+^ reabsorption by increasing both Na^+^ affinity and *V*_max_ of Na,K‐ATPase at the basolateral membrane, thus increasing the driving force for Na^+^ entry across the apical membrane. However, the kidney is apparently well‐adapted to match Na^+^ output to Na^+^ intake, and a renal phenotype is very mild (Arystarkhova et al. 2013; Jones et al. 2005). Under resting conditions, no significant differences between genotypes were seen in plasma concentration of Na^+^ or in plasma osmolality (Jones et al. 2005). We found a higher concentration of Na^+^ and higher osmolality in urine from the knockout mice with 24 h collection in metabolic cages, however, a slight but statistically significant reduction in urine output apparently compensated the total excretion of Na^+^ (Arystarkhova et al. 2013).

FXYD2 has a highly restricted distribution in the body and was originally thought to be present only in the kidney, but we and others discovered that it is also expressed in pancreatic beta cells (Arystarkhova et al. 2013; Flamez et al. 2010). The knockout mice have a metabolic phenotype of low glucose and twofold elevated fasting plasma insulin linked to beta cell hyperplasia (Arystarkhova et al. 2013). Insulin is antinatriuretic and should further activate renal Na,K‐ATPase (Tiwari et al. 2007), however, and so the knockout's metabolic phenotype does not suggest a mechanism for the observed renal adaptation.

We hypothesized that the renal adaptation observed in the *Fxyd2*^*−/−*^ mice may involve compensatory reductions in luminal Na^+^ uptake. Here, we focused on distal convoluted tubule (DCT). It is the segment with the highest level of expression of the Na,K‐ATPase in kidney, and it is known for dramatic compensatory plasticity, including not only regulation and expression changes of transporters but also cellular hyperplasia and hypoplasia (Subramanya and Ellison 2014). We report that in mice with global deletion of *Fxyd2* there was evidence for marked stimulation of the thiazide‐sensitive NCC cotransporter. This seems paradoxical because NCC activation is expected to increase Na^+^ retention and is often associated with an increase in arterial blood pressure (Hoorn et al. 2011; Moes et al. 2014; Gamba 2005), a symptom that was not observed in *Fxyd2*^*−/−*^ mice.

## Materials and Methods

### Animals

All procedures involving mice were carried out using protocols approved by the Massachusetts General Hospital Subcommittee on Research Animal Care and in accordance with the National Institutes of Health's *Guide for the Care and Use of Laboratory Animals*. *Fxyd2*^*−/−*^ mice (Fxyd2^tm1Kdr^) were used from the 9^th^ to the 17^th^ backcross to the C57BL/6NCrl mouse strain. Each generation of mice for experiments was produced from heterozygote parents that resulted from back‐crosses to fresh C57Bl/6N wild types obtained from Charles River Laboratories, Wilmington, MA. Offspring were genotyped by PCR amplification of ear punch DNA taken at weaning. Mice were given regular diet (0.3% Na^+^; ProLab IsoPro RMH 3000 [PMI Nutrition International, LLC, Brentwood, MO]) and had free access to water on a 12‐h dark/light cycle.

### Laboratory tests

Plasma electrolytes (Na^+^, K^+^, and Cl^−^), were measured with an Instat system blood analyzer (Abbott, Princeton, NJ). Na^+^ in urine was measured at IDEXX Preclinical Research Labs with a DX Chemistry Analyzer.

### Antibodies

Rabbit antisera K1 or K3 were used to detect *α*1 and *β*1 subunits of Na,K‐ATPase on blots, as described elsewhere (Sweadner and Gilkeson 1985). The RCT‐G1 polyclonal antibody raised against the shared FXYD2 COOH‐terminal peptide and FXYD2b N‐terminus‐specific polyclonal antibody was described previously (Arystarkhova et al. 1999, 2002b). Monoclonal antibody BSP3 (a kind gift from Dr. C. Goridis, Centre d'Immunologie de Marseille‐Luminy, France) was employed for detection of the *β*1‐subunit of Na,K‐ATPase on sections. Antibodies against total and phosphorylated forms of NCC, as well as total NHE3 exchanger, were described previously (Nguyen et al. 2013). Total and phosphorylated species of NKCC2 were probed with polyclonal antibodies kindly provided by Dr. K. Mutig (Charité‐Universitätsmedizin Berlin, Berlin, Germany). Detection of full length and cleaved forms of *α*‐ENaC, as well as phosphorylated SPAK (pS373), were as reported previously with a LiCor Odyssey system (Nguyen et al. 2013).

### Membrane preparations and gel analysis

Crude membrane preparations for gel electrophoresis were obtained from superficial renal cortex by homogenization in a buffer containing 250 mmol/L sucrose, 1 mmol/L EDTA, and 10 mmol/L Tris, pH 7.4, and differential centrifugation at 3000 ×*g*, 15 min, at 4°C (Sorvall, SS‐34), followed by centrifugation of the supernatant at 100,000 ×*g*, 60 min, at 4°C (Beckman [Indianapolis, IN], Ti 70.5). Final pellets were resuspended in a buffer containing 250 mmol/L sucrose, 1 mmol/L EDTA and 10 mmol/L Tris, pH 7.4. Proteins were resolved on 4–12% NuPage MES‐SDS gels (Life Technologies, Grand Island, NY), transferred to nitrocellulose, and incubated with specific antibodies. Detection was with chemiluminescence using a digital imaging system, ImageQuant LAS4000 (GE Healthcare Biosciences, Pittsburgh, PA). Quantification was with ImageQuant TL image analysis software.

### Enzymatic assays

Total Na,K‐ATPase activity was measured in media containing 100 mmol/L NaCl, 20 mmol/L KCl, 3 mmol/L Tris‐ATP, 3 mmol/L MgCl_2_, 30 mmol/L histidine, pH 7.4, on the same crude membrane preparations, with no additional stimulation with a detergent. All of the reactions were performed at 37°C for 30 min with and without 3 mmol/L ouabain, and ouabain‐sensitive P_i_ release was measured colorimetrically by the Fiske–Subbarow method (Arystarkhova et al. 2002a). Data were analyzed by GraphPad Prism 6 software (GraphPad Software, La Jolla, CA).

### Immunofluorescence

Cryostat sections (5 μm thickness) of paraformaldehyde/lysine/periodate‐fixed kidneys (2% PLP) were treated with 1% SDS in phosphate saline buffer for antigen retrieval (Brown et al. 1996), and then dual stained with rabbit antibodies anti‐pT53 or anti‐pS71, against phosphorylated forms of NCC transporter, in combination with monoclonal antibody BSP3 against Na,K‐ATPase *β*1. Detection was with Alexa Fluor‐conjugated secondary antibodies (Life Technologies). Images were collected on a Zeiss LSM Pascal 5 scanning laser confocal system.

### Blood pressure measurement

The CODA 4‐Channel Non‐Invasive Blood Pressure tail‐cuff system (Kent Scientific, Torrington, CT), was used to measure the blood pressure in up to four mice simultaneously. Animals were acclimated and trained for four consecutive days prior to recording experimental data. The procedure was performed in accordance with the manufacturer's manual.

### Acute saline challenge

To assess the rate at which a saline load is excreted, WT and knockout mice were injected i.p. with a volume of 0.9% saline equivalent to 10% of their body weight, and were placed immediately in metabolic cages for urine collection. Results are expressed as the fraction of the saline load excreted over 4 h. The rate of excretion should depend inversely on sodium transporter activation along the nephron.

### Statistical analysis

Results were analyzed with unpaired Student's *t*‐test and were expressed as means of 4–6 independent experiments ± SEM. A two‐tailed *P* value < 0.05 was considered significant.

## Results

### Na,K‐ATPase in cortex

FXYD2 is an endogenous inhibitory subunit of Na,K‐ATPase which is expressed abundantly in PT, MTAL, proximal CTAL, DCT, CNT, and lightly in inner medullary collecting duct. Thus, a renal phenotype was expected in the knockout animal. However, no significant differences between wild‐type and FXYD2‐depleted mice were found in plasma concentration of major electrolytes or plasma osmolality under basal conditions (Table 1).

**Table 1.** Major electrolytes and osmolality in plasma from wild‐type and *Fxyd2*^−/−^ mice.

	Wild type	*Fxyd2* ^*−/−*^	Statistical significance
Na^+^, mmol/L	141.9 ± 2.4	143.2 ± 3.4	*P* = 0.76
K^+^, mmol/L	5.0 ± 0.26	5.04 ± 0.3	*P* = 0.93
Cl^−^, mmol/L	115.0 ± 2.7	116.1 ± 3.6	P = 0.80
Plasma osmolality, mOsm	311 ± 3.7	310 ± 8.1	*P* = 0.89

Data were analyzed by unpaired *t*‐test and presented as means ± SEM. For potassium analysis, mice were fasted overnight. *N* = 6–8 for each genotype.

**Figure 1.**
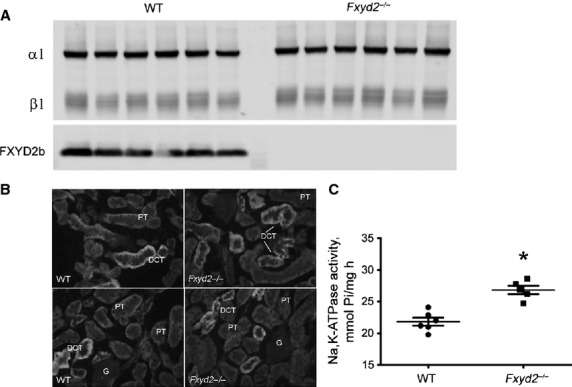
Na, K‐ATPase in renal cortex from WT and *Fxyd2*^−/−^ mice. (A). Representative Western blot shows staining with K3 antibody to detect Na,K‐ATPase *α*1 and *β*1 subunits in cortical membranes. Equal amounts of protein (5 μg) were loaded per lane, *n* = 6 for each genotype. Staining with antibody against FXYD2b (RNGB) was used to verify mouse genotype. (B) Immunofluorescence staining forβ *β*1 subunit of Na,K‐ATPase. PT, proximal convoluted tubules, DCT, distal convoluted tubules, G, glomerulus. Intensity of stain was higher in DCT than in PT, and similar for both genotypes. (C) Na,K‐ATPase activity was assayed from membranes of WT (closed circles) and *Fxyd2*^−/−^ mice (closed squares) (*n* = 6 for each genotype). Ouabain‐dependent ATP hydrolysis (*V*_max_, μmol Pi/mg h) is expressed as means ± SEM. The asterisk indicates statistical significance (*P* < 0.05).

The first question was whether biosynthesis of the Na,K‐ATPase itself was adaptively modulated in *Fxyd2*^*−/−*^ mice to compensate for loss of the inhibitory subunit. Figure 1A demonstrates Western blot analysis of crude membrane preparations from renal cortex of WT and *Fxyd2*^*−/−*^ mice. Blots were stained with the K3 antiserum, and both *α*1 and *β*1 subunits of the Na,K‐ATPase were quantified. Comparison of WT and knockout mice showed no statistically significant difference in the abundance of either subunit. Similar results were obtained with another antibody against *α*1 (not shown). There was also no change in expression of NHE3 [1.0 ± 0.15 vs. 0.99 ± 0.19 (*P* = 0.99), not shown]. Thus, global deletion of FXYD2 did not change total expression of Na,K‐ATPase in renal cortex. Staining with anti‐FXYD2b is presented for verification of the knockout animals.

Since membrane preparations used for blots contained a mixture of cortical nephron segments – proximal tubules, distal convoluted tubules, connecting tubules, cortical thick ascending limb, and cortical collecting duct – immunocytochemistry was performed to monitor relative expression of Na,K‐ATPase in *Fxyd2*^−/−^ mice. Figure 1B shows representative images of immunostaining for *β*1 subunit. No significant difference in pattern and relative intensity of staining was observed between genotypes. Higher magnification confocal data are shown for *β*1 below, and similar results were obtained with an antiserum against *α*1 subunit (not shown).

While no difference in the abundance and relative distribution of Na,K‐ATPase was seen in renal cortex, in membranes we assayed a statistically significant 1.25‐fold increase in ouabain‐dependent ATP hydrolysis activity of Na,K‐ATPase from knockout mice compared to WT: 26.8 ± 1.5 vs. 21.4 ± 1.2 μmol P_i_/mg/h, respectively (*P* < 0.001, *n* = 6 for each genotype) (Fig. 1C). The data are in agreement with the previously reported role of FXYD2 as an endogenous inhibitory subunit of the Na,K‐ATPase. It should be noted that reactions were performed in reaction medium with saturating [Na^+^], that is, the difference in activity reflects changes at the *V*_max_ level.

### NCC in Fxyd2^*−/−*^ mice

**Figure 2.**
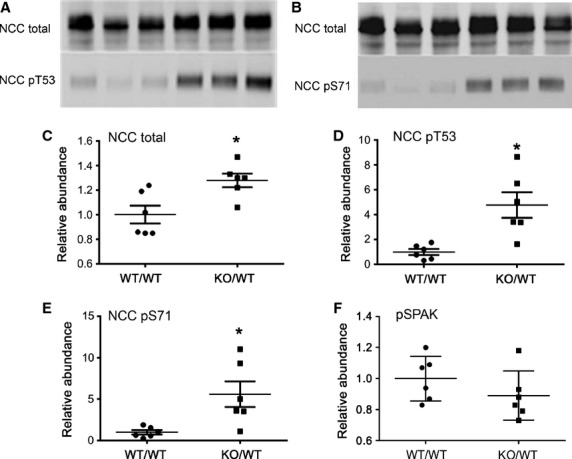
Enhanced abundance and basal NCC phosphorylation in *Fxyd2*^−/−^ mice. Representative immunoblots of NCC total, NCCpT53 and NCC pS71 in renal cortex of WT and *Fxyd2*^−/−^ mice (A and B). Equal amounts of protein were loaded per lane. Blots were scanned, and the density values were normalized to mean density of the WT group for NCC total (C), NCC pT53 (D), NCC pS71 (E), and pSPAK (F).The data are expressed as means ± SEM. Asterisks indicate statistical significance (*P* < 0.01).

**Figure 3.**
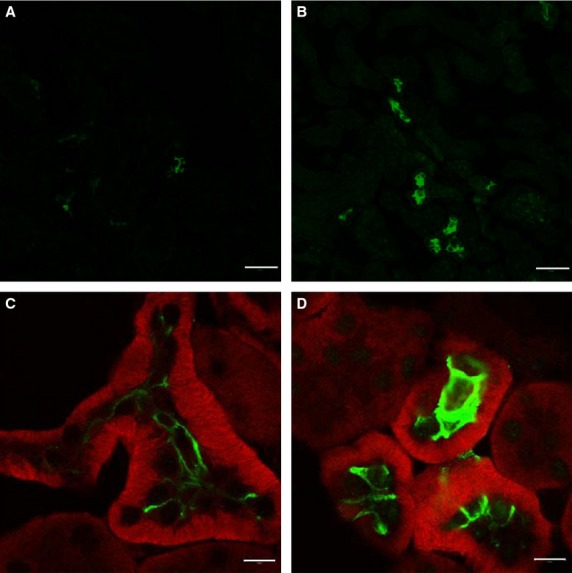
Immunofluorescence localization of phosphorylated forms of NCC in kidneys from WT and *Fxyd2*^−/−^ mice. (A and B). Staining with anti‐NCC pS71 antibody on sections from WT (A) and *Fxyd2*^−/−^ (B) mice. While almost no signal was detected in WT (A), a significant increase in phosphorylation at Ser71 was seen in apical DCT membranes in kidney from knockout mice (B). Bar, 50 μm. (C and D). Dual immunofluorescent staining for *β*1 subunit of Na,K‐ATPase (red) and anti‐NCC pS71 (green) is shown at high magnification. No significant difference was noticed in the expression level of Na,K‐ATPase *β*1 subunit in DCT between WT and *Fxyd2*^−/−^ mice, while great enhancement was seen in the phosphorylation level of NCC at Ser71 in the knockout mice (D) compared to WT (C). Bar, 10 μm.

The thiazide‐sensitive Na^+^‐Cl^−^ transporter, NCC, is expressed exclusively in the DCT (Gamba 2012). It is the principal candidate for adaptive regulation of Na^+^ retention in the distal tubule because it is paired with the highest level of Na,K‐ATPase in the kidney. Figure 2 A and B show representative Western blots of cortical membranes from WT and *Fxyd2*^*−/−*^ mice stained for total and phosphorylated forms of the NCC cotransporter (five males and one female were used for each genotype). Statistical analysis revealed a 1.28 ± 0.06 fold increase in the abundance of total NCC cotransporter in the knockout over WT mice (*P* < 0.05, *n* = 6 for each genotype) (Fig. 2C). This increase correlated well with the enhanced activity of Na,K‐ATPase in cortex from the *Fxyd2*^*−/−*^ mice described above. Additionally, analysis of phosphorylated NCC species revealed a much greater difference: 4.8 ± 1.0 and 5.6 ± 1.5 fold increase in knockout over wild‐type mice for phosphorylation at T53 and S71 residues (Fig. 2D and E, respectively; *P* < 0.01). The phosphorylated form of NCC is localized exclusively at the plasma membrane (Lee et al. 2013). To assess the localization and verify the difference in NCC phosphorylation between WT and *Fxyd2*^*−/−*^ mice shown above, cryosections (5 μm) from PLP‐fixed kidneys were stained for pS71 NCC. WT mice displayed only light apical phosphorylation at Ser71 (Fig. 3A), whereas it was greatly enhanced in kidney from knockout mice (Fig. 3B). Figure 3C and D show high magnification images with dual immunostaining of DCT for *β*1 subunit of Na,K‐ATPase (red) and pS71 NCC (green). While staining intensity for basolateral Na,K‐ATPase was similar in WT and *Fxyd2*^*−/−*^ mice, there was a significant increase in apical pS71 NCC in DCT from *Fxyd2*^*−/−*^ (Fig. 3D) over WT mice (Fig. 3C). Similar results were obtained with anti‐pT53 NCC antibody (not shown). The data are in agreement with Western blot analysis and suggest baseline activation of NCC cotransporter in kidney from *Fxyd2*^*−/−*^ mice.

### Lack of DCT morphological change

As seen in Figs. 1B, 3C and D, the cellular morphology of the DCT cells, stained for Na,K‐ATPase *β*1 subunit, was not obviously altered in the knockout.

### NKCC2 in cTAL of Fxyd2^*−/−*^ mice

**Figure 4.**
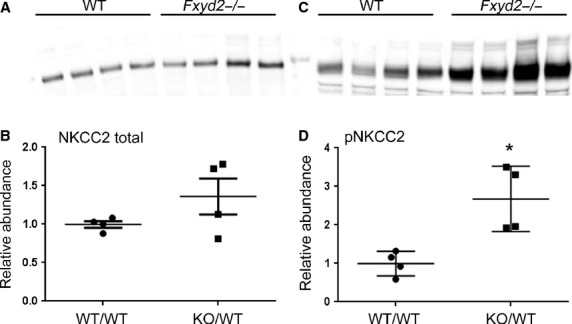
Basal expression and phosphorylation of NKCC2 transporter. Cortical membrane fractions from WT and *Fxyd2*^−/−^ mice were analyzed by Western blots with the antibodies against total (A) and phosphorylated NKCC2 (C). Blots were scanned and the density values were normalized to mean density of the WT group for total (B) and pNKCC2 (D). The data are expressed as means ± SEM. Asterisk indicates statistical significance (*P* < 0.01).

We analyzed whether reduced activity of NKCC2, (SLC12A1), located upstream in thick ascending limb, might drive a compensatory activation of NCC in DCT. Contrary to this hypothesis, Fig. 4 demonstrates in samples of renal cortex that there was no significant difference in total NKCC2 between genotypes: 1.0 ± 0.04 versus 1.36 ± 0.23 (*n* = 4, mean ± SEM, *P* = 0.18) for WT and knockout. Furthermore, phosphorylated NKCC2 was significantly higher in the knockouts, evidence instead for activation: 1.0 ± 0.16 versus 2.68 ± 0.43 (*n* = 4, mean ± SEM, *P* = 0.01). The data suggest that both NCC in DCT and NKCC2 in CTAL are activated in *Fxyd2*^*−/−*^ mice under basal conditions. Phosphorylation of NCC and NKCC2 are often mediated by the SPAK (STE‐20 related proline/alanine‐rich kinase) pathway (Gamba 2012). Surprisingly, no increase in phosphorylation of the SPAK kinase was observed in knockout mice based on unpaired t‐test (Fig. 2E): 1.0 ± 0.06 versus 0.89 ± 0.06 (mean ± SEM, *P* = 0.24). Whether the greater phosphorylation of NKCC2 and NCC in *Fxyd2*^*−/−*^ mice occurs via recently identified SPAK‐OSR1‐independent pathways (Ponce‐Coria et al. 2014), or via reduced rates of transporter dephosphorylation, remains to be determined.

### ENaC in Fxyd2^*−/−*^ mice

To determine whether the activation of NKCC2 and NCC was a compensatory response to reduced Na^+^ reabsorption further along the nephron in *Fxyd2*^*−/−*^ mice, expression of full length as well as proteolytically cleaved (activated) *α*‐ENaC was determined by immunoblot. Statistical analysis revealed no significant changes between genotypes: 1.0 ± 0.09 (WT) versus 0.82 ± 0.07 (Fxyd2^*−/−*^), *P* = 0.14, and 1.0 ± 0.1 (WT) versus 0.88 ± 0.09 (*Fxyd2*^*−/−*^), *P* = 0.39, for total and cleaved forms, respectively. The data do not support the notion that depressed ENaC contributed to the phenomenon of hyperstimulation of NCC transporter without evident Na^+^ retention.

### Blood pressure

**Figure 5.**
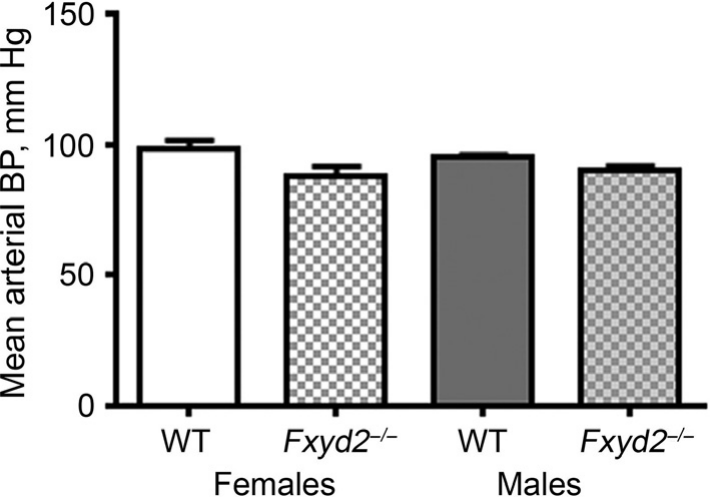
Increased expression and phosphorylation of NCC do not increase arterial blood pressure in *Fxyd2*^−/−^ mice under basal conditions. Males (gray background) and females (white background) of WT (open boxes) and knockout mice (patterned boxes) (*n* = 5–6 for each genotype) were subjected to blood pressure measurement by the tail‐cuff method. At least 10–20 cycles were averaged for each measurement. Data were analyzed by unpaired Student's *t*‐test producing two‐tailed *P* values < 0.001 and < 0.05 for males and females, respectively. The data show a slight reduction, not the predicted increase, in pressure. Data are presented as the means ± SEM.

Phosphorylation of NCC at Thr53 and Ser71 residues has been previously associated with activation of NCC cotransporter and development of hypertension (Hoorn et al. 2011). We tested whether *Fxyd2*^*−/−*^ mice have elevated blood pressure compared to WT using the tail‐cuff noninvasive technique. As shown in Fig. 5, neither male nor female FXYD2 knockout mice exhibited blood pressure elevation. Moreover, there was a slight reduction in mean arterial pressure in knockout compared to WT mice: males 87 ± 2 versus 95 ± 1 and females 87 ± 4 versus 98 ± 4, respectively. Although statistical significance was reached for both genders (two‐tailed *P* value < 0.05), there was a large variation in measurements. Thus, the conservative conclusion is that in the *Fxyd2*^*−/−*^ mice, activation of NCC, evident as increased abundance of NCC‐P, was not associated with development of hypertension.

### Acute saline challenge

**Figure 6.**
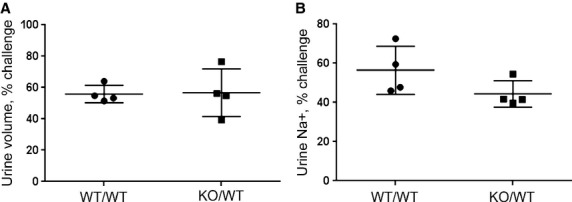
Acute sodium challenge did not reveal difference in Na^+^ retention between WT and *Fxyd2*^−/−^ mice. Animals were loaded with saline (i.p., 10% of the weight) and put in metabolic cages for urine collection for 4 h. Results are expressed as urine volume (A) or urine Na^+^ (B) as a percent of the load excreted in 4 h. Data are presented as means ± SEM.

To assess whether the increases in NCC‐P and NKCC2‐P in knockouts were associated with Na^+^ retention, we analyzed sodium and volume excretion in the first 4 h after an acute saline load. As shown in Fig. 6, no statistical difference was noted between WT and *Fxyd2*^*−/−*^ mice in the percent of load in urine volume, 55.9 ± 2.8% versus. 56.8 ± 7.6% (*P* = 0.91), or urine Na^+^, 56.5 ± 6.2% versus 44.4 ± 3.4% (*P* = 0.14). The data suggest that there is no increase in sodium reabsorption in *Fxyd2*^*−/−*^ mice despite hyperphosphorylation of NKCC2 and NCC.

## Discussion

Total Na,K‐ATPase activity was elevated 1.25‐fold in cortical membranes from *Fxyd2*^*−/−*^ mice, which should in principle result in enhanced sodium retention and altered urine composition. In practice, however, the mouse was well balanced. Analysis of plasma electrolytes (Na^+^, K^+^, and Cl^−^) and plasma osmolality did not reveal any significant difference between genotypes (Table 1). Similarly, in our previous work no difference was seen in urine electrolytes (Na^+^, K^+^, and Mg^2+^). Although we detected a higher concentration of Na^+^ and higher osmolality in urine from KO mice, total Na^+^, and mOsmol excreted per day was compensated by a slightly reduced urine volume (Arystarkhova et al. 2013). In principle, Na^+^ retention should put the animal at risk for hypertension, but instead the *Fxyd2*^*−/−*^ mouse has normal blood pressure. This along with the sodium balance implies either that NaCl uptake is adaptively restricted, or that NaCl efflux is facilitated in appropriate renal segments.

DCT is the segment with the highest density and activity of Na,K‐ATPase. If this particular segment is involved in compensation, luminal sodium uptake should be reduced, that is, activity of NCC transporter should be diminished. Instead we observed up regulation of NCC protein expression and highly augmented phosphorylation at Thr53 and Ser71 sites. The latter is particularly interesting since phosphorylation of a cluster of Ser/Thr residues in the amino‐terminal domain of NCC has been shown to correlate with activation of this cotransporter, and is associated with elevation of blood pressure (Gamba 2012). For example, NCC‐overexpressing mice with no phospho‐NCC at baseline showed no hypertension, but phospho‐activation of NCC with a synthetic mineralocorticoid produced hypertension similar to WT (McCormick et al. 2011). Thus, activity of NCC in *Fxyd2*^*−/−*^ mice is predicted to be significantly augmented compared to WT. An enhancement in apical Na^+^ entry in DCT cells, however, would not explain the compensated physiology of the mouse and lack of hypertension (Subramanya and Ellison 2014).

There are several physiological triggers associated with NCC phosphorylation, such as chronic Ang II or aldosterone infusion (Castañeda‐Bueno et al. 2012), acute vasopressin treatment (Mutig et al. 2010), insulin treatment (Sohara et al. 2011; Gamba 2012), and low‐salt diet or hypovolemia, which activate the renin–angiotensin system (Chiga et al. 2008; Vallon et al. 2009). Low dietary K^+^ also activates NCC, and high dietary K^+^ inhibits it, possibly to regulate the Na^+^ supply for downstream K^+^ pathways (Castañeda‐Bueno et al. 2014; Rengarajan et al. 2014). *Fxyd2*^*−/−*^ mice are neither hypovolemic (based on Echo‐MRI analysis, not shown), nor hypokalemic (Table 1). However, *Fxyd2*^*−/−*^ mice do have a higher level of circulating insulin than WT (Arystarkhova et al. 2013). Whether insulin plays a role in hyperphosphorylation of NCC in *Fxyd2*^*−/−*^ under basal conditions awaits further investigation. Nevertheless, all of the physiological states mentioned above call for, or cause, an increase in Na^+^ retention, and are the opposite of an expected adaptive response to excess Na^+^ retention. Phosphorylation of NCC is normally mediated by the WNK (with no lysine kinase)‐SPAK (STE‐20 related proline/alanine‐rich kinase) pathways (Gamba 2012). Here, however, there was no increase in phosphorylation of SPAK in knockout mice. This would imply a SPAK‐independent mechanism of regulation of NCC activity.

Is phosphorylation of NCC in *Fxyd2*^*−/−*^ mice adaptive? Theoretically it could be if NCC transporter operated in a reverse mode and released Na^+^ into the lumen. However, this would require ion gradients between cell and lumen that are unlikely to be achieved physiologically. Alternatively, phosphorylation of NCC in *Fxyd2*^*−/−*^ mice could be only locally adaptive, resulting from activation of cell‐autonomous pathways that normally match the size of apical and basolateral transport fluxes so that transepithelial transport occurs without major changes in cytoplasmic ion concentrations [e.g., (Wang et al. 2014)]. If so, the adapted state of the *Fxyd2*^*−/−*^ mice would have to be a consequence of effective counteradaptations in other nephron segments.

Adaptation may entail regulation of other apical transporters (Hadchouel et al. 2010; Wang et al. 2014). Reduction in NKCC2 in TAL would be compensatory, however, we observed twofold phosphoactivation of cortical NKCC2 transporter in knockout mice. In principle, NCC could be high in *Fxyd2*^*−/−*^ mice to compensate for low ENaC activity in the late DCT (Hadchouel et al. 2010), but that also was not the case here. If ENaC activity was low, there would be less ENaC *α* subunit cleavage, and probably less ENaC total. Our data thus also rule out late distal nephron as a site for compensation for an increase in Na,K‐ATPase activity. One might also hypothesize that hyperactivity of NCC in the DCT of *Fxyd2*^*−/−*^ would reduce Na^+^ delivery to the CCD where it drives K^+^ secretion, thus reducing K^+^ secretion (McDonough and Youn 2013). This could be relevant if plasma K^+^ was low in *Fxyd2*^*−/−*^, but we did not observe significant differences between genotype in plasma electrolytes (Table 1).

The increased activity of the Na,K‐ATPase in the knockout mice might be expected to increase the fraction of the filtered sodium load absorbed in the proximal nephron. As a result, the amount delivered to the macula densa could be decreased. FXYD2 is also normally expressed in macula densa (Pu et al. 2001; Wetzel and Sweadner 2001, 2003), however, and the effects of its absence on juxtaglomerular apparatus function, positive or negative, are not yet known. Hypothetically, enhanced Na,K‐ATPase activity due to FXYD2 absence would promote macula densa cell shrinkage and participate positively in the cascade of events that release renin. Thus, genetic deletion of FXYD2 may also impact the macula densa in a maladaptive way.

Finally, the increased Na^+^ uptake caused by elevated Na,K‐ATPase activity in *Fxyd2*^*−/−*^ mice in principle may be counteracted mainly in the proximal tubule. Such regulation could be either a cause of, or a response to, the hyperactivation of NCC and NKCC2, as follows. The goal of PT adaption to the absence of FXYD2 would be to bring elevated PT reabsorption of sodium back down to 65% of load, like WT. Reducing the activity of apical NHE3, which is thought to normally be rate‐limiting, would limit reabsorption (McDonough 2010), and locally produced dopamine might coordinate the reduction in apical transporters and Na,K‐ATPase (Aperia 2000; Armando et al. 2011). This could be via trafficking transporters out of the membrane without change in protein abundance (McDonough 2010; Chen et al. 2009). If these available PT adaptive mechanisms could not exactly match the genetic Na,K‐ATPase increase within PT, a net natriuretic effect could elicit the downstream activation of NKCC2 and NCC to bring the animal into sodium balance. Alternatively, if the activation of NCC and NKCC2 was instead due to intracell coupling of apical and basolateral membrane function, producing a maladaptive excess reabsorption, that could also in principle be counteracted by more robust inhibition of uptake in proximal tubules. The observation that acute sodium loading did not reduce sodium excretion is consistent with both scenarios if adaptive mechanisms are not maxed out in the *Fxyd2*^*−/−*^ mouse.

Further experiments employing modulation of diet and diuretic states might unveil phenotypic differences between genotypes. If the observed activation of NCC and NKCC2 in *Fxyd2*^*−/−*^ mice is adaptive to compensate Na^+^ wasting in proximal tubules, diuretic challenge should reveal higher Na^+^ excretion in knockout animals. On the other hand, in order to avoid Na^+^ overload under high sodium challenge, later nephron segments would have to switch from Na^+^ retention to natriuresis mode. Thus, there should be a reduction in NCC and NKCC2 phosphorylation even in the *Fxyd2*^*−/−*^ mouse.

Regardless of the mechanism of adaptation and where it is localized in the nephron, the *Fxyd2*^*−/−*^ knockout mouse is in a desirable compensated state, and so it potentially opens a window into a mechanism that can compensate or override the hypertension often correlated with NCC activation. The response of NCC to this genetic perturbation contrasts strikingly with the downregulation and dephosphorylation of NCC seen in response to elevated dietary or infused K^+^, which produces natriuresis in rats (Rengarajan et al. 2014).

## Acknowledgments

The authors appreciate stimulating discussions with Dr. Dennis Brown (Program of Membrane Biology, Massachusetts General Hospital), and Dr. S.J.D. Karlish (Weizmann Institute). We are grateful to Dr. Ana Dordea and Dr. Emmanuel Buys for technical assistance with the CODA Blood Pressure measurement instrument.

## Conflict of Interest

No conflicts of interest, financial or otherwise, are declared by the authors

## References

[b1] Aperia A. 2000 Intrarenal dopamine: a key signal in the interactive regulation of sodium metabolism. Annu. Rev. Physiol.; 62:621-647. 10845105 10.1146/annurev.physiol.62.1.621

[b2] Armando I. Villar V. A. Jose P. A. 2011 Dopamine and renal function and blood pressure regulation. Compr. Physiol.; 1:1075-1117. 23733636 10.1002/cphy.c100032 PMC6342207

[b3] Arystarkhova E. Wetzel R. K. Asinovski N. K. Sweadner K. J. 1999 The γ subunit modulates Na^+^ and K^+^ affinity of the renal Na, K‐ATPase. J. Biol. Chem.; 274:33183-33185. 10559186 10.1074/jbc.274.47.33183

[b4] Arystarkhova E. Donnet C. Asinovski N. K. Sweadner K. J. 2002a Differential regulation of renal Na, K‐ATPase by splice variants of the γ subunit. J. Biol. Chem.; 277:10162-10172. 11756431 10.1074/jbc.M111552200

[b5] Arystarkhova E. Wetzel R. K. Sweadner K. J. 2002b Distribution and oligomeric association of splice forms of the Na, K‐ATPase regulatory γ subunit in rat kidney. Am. J. Physiol.; 282:F393-F407. 10.1152/ajprenal.00146.2001 11832419

[b6] Arystarkhova E. Liu Y. B. Salazar C. Stanojevic V. Clifford R. J. Kaplan J. H. 2013 Hyperplasia of pancreatic beta cells and improved glucose tolerance in mice deficient in FXYD2. J. Biol. Chem.; 288:7077-7085. 23344951 10.1074/jbc.M112.401190 PMC3591617

[b7] Brown D. Lydon J. McLaughlin M. Tilly A. S. Tyszkowski R. Alper S. 1996 Antigen retrieval in cryostat tissue sections and cultured cells by treatment with sodium dodecyl sulfate (SDS). Histochem. Cell Biol.; 105:261-267. 9072183 10.1007/BF01463929

[b8] Capurro C. Coutry N. Bonvalet J. P. Escoubet B. Garty H. Farman N. 1996 Cellular localization and regulation of CHIF in kidney and colon. Am. J. Physiol.; 271:C753-C762. 8843704 10.1152/ajpcell.1996.271.3.C753

[b9] Castañeda‐Bueno M. Cervantes‐Pérez L. G. Vázquez N. Uribe N. Kantesaria S. Morla L. 2012 Activation of the renal Na^+^:Cl^−^ cotransporter by angiotensin II is a WNK4‐dependent process. Proc. Natl Acad. Sci. USA; 109:7929-7934. 22550170 10.1073/pnas.1200947109 PMC3356635

[b10] Castañeda‐Bueno M. Cervantes‐Pérez L. G. Rojas‐Vega L. Arroyo‐Garza I. Vázquez N. Moreno E. 2014 Modulation of NCC activity by low and high K^+^ intake: insights into the signaling pathways involved. Am. J. Physiol. Renal. Physiol.; 306:F1507-F1519. 24761002 10.1152/ajprenal.00255.2013 PMC4059971

[b11] Chen Z. Leibiger I. Katz A. I. Bertorello A. M. 2009 Pals‐associated tight junction protein functionally links dopamine and angiotensin II to the regulation of sodium transport in renal epithelial cells. Br. J. Pharmacol.; 158:486-493. 19563532 10.1111/j.1476-5381.2009.00299.x PMC2757688

[b12] Chiga M. Rai T. Yang S. S. Ohta A. Takizawa T. Sasaki S. 2008 Dietary salt regulates the phosphorylation of OSR1/SPAK kinases and the sodium chloride cotransporter through aldosterone. Kidney Int.; 74:1403-1409. 18800028 10.1038/ki.2008.451

[b13] Flamez D. Roland I. Berton A. Kutlu B. Dufrane D. Beckers M. C. 2010 A genomic‐based approach identifies FXYD domain containing ion transport regulator 2 (FXYD2)γa as a pancreatic beta cell‐specific biomarker. Diabetologia; 53:1372-1383. 20379810 10.1007/s00125-010-1714-z

[b14] Gamba G. 2005 Molecular physiology and pathophysiology of electroneutral cation‐chloride cotransporters. Physiol. Rev.; 85:423-493. 15788703 10.1152/physrev.00011.2004

[b15] Gamba G. 2012 Regulation of the renal Na^+^‐Cl^−^ cotransporter by phosphorylation and ubiquitylation. Am. J. Physiol. Renal. Physiol.; 303:F1573-F1583. 23034942 10.1152/ajprenal.00508.2012 PMC3532472

[b16] Geering K. 2006 FXYD proteins: new regulators of Na‐K‐ATPase. Am. J. Physiol.; 290:F241-F250. 10.1152/ajprenal.00126.2005 16403837

[b17] Hadchouel J. Soukaseum C. Büsst C. Zhou X. Baudrie V. Zürrer T. 2010 Decreased ENaC expression compensates the increased NCC activity following inactivation of the kidney‐specific isoform of WNK1 and prevents hypertension. Proc. Natl Acad. Sci. USA; 107:18109-18114. 20921400 10.1073/pnas.1006128107 PMC2964238

[b18] Hoorn E. J. Walsh S. B. McCormick J. A. Fürstenberg A. Yang C. L. Roeschel T. 2011 The calcineurin inhibitor tacrolimus activates the renal sodium chloride cotransporter to cause hypertension. Nat. Med.; 17:1304-1309. 21963515 10.1038/nm.2497 PMC3192268

[b19] Jones D. H. Li T. Y. Arystarkhova E. Barr K. J. Wetzel R. K. Peng J. 2005 Na, K‐ATPase from mice lacking the γ subunit (FXYD2) exhibits altered Na^+^ affinity and decreased thermal stability. J. Biol. Chem.; 280:19003-19011. 15755730 10.1074/jbc.M500697200

[b20] Küster B. Shainskaya A. Pu H. X. Goldshleger R. Blostein R. Karlish S. J. D. 2000 A new variant of the γ subunit of renal Na, K‐ATPase. Identification by mass spectrometry, antibody binding and expression in cultured cells. J. Biol. Chem.; 275:18441-18446. 10748024 10.1074/jbc.M001411200

[b21] Lee D. H. Maunsbach A. B. Riquier‐Brison A. D. Nguyen M. T. X. Fenton R. A. Bachmann S. 2013 Effects of ACE inhibition and ANG II stimulation on renal Na‐Cl cotransporter distribution, phosphorylation, and membrane complex properties. Am. J. Physiol. Cell Physiol.; 304:C147-C163. 23114965 10.1152/ajpcell.00287.2012 PMC3587378

[b22] Lubarski I. Pihakaski‐Maunsbach K. Karlish S. J. D. Maunsbach A. B. Garty H. 2005 Interaction with the Na, K‐ATPase and tissue distribution of FXYD5 (RIC). J. Biol. Chem.; 280:37717-37724. 16148001 10.1074/jbc.M506397200

[b23] McCormick J. A. Nelson J. H. Yang C. L. Curry J. N. Ellison D. H. 2011 Overexpression of the sodium chloride cotransporter is not sufficient to cause familial hyperkalemic hypertension. Hypertension; 58:888-894. 21896937 10.1161/HYPERTENSIONAHA.110.167809 PMC3199361

[b24] McDonough A. A. 2010 Mechanisms of proximal tubule sodium transport regulation that link extracellular fluid volume and blood pressure. Am. J. Physiol. Regul. Integr. Comp. Physiol.; 298:R851-R861. 20106993 10.1152/ajpregu.00002.2010 PMC2853398

[b25] McDonough A. A. Youn J. H. 2013 Need to quickly excrete K^+^? Turn off NCC. Kidney Int.; 83:779-782. 23633048 10.1038/ki.2012.468 PMC3644996

[b26] Moes A. D. van der Lubbe N. Zietse R. Loffing J. Hoorn E. J. 2014 The sodium chloride cotransporter SLC12A3: new roles in sodium, potassium, and blood pressure regulation. Pflug. Arch.; 466:107-118. 10.1007/s00424-013-1407-9 24310820

[b27] Mutig K. Saritas T. Uchida S. Kahl T. Borowski T. Paliege A. 2010 Short‐term stimulation of the thiazide‐sensitive Na^+^‐Cl^−^ cotransporter by vasopressin involves phosphorylation and membrane translocation. Am. J. Physiol. Renal. Physiol.; 298:F502-F509. 20007345 10.1152/ajprenal.00476.2009

[b28] Nguyen M. T. X. Lee D. H. Delpire E. McDonough A. A. 2013 Differential regulation of Na^+^ transporters along nephron during ANG II‐dependent hypertension: distal stimulation counteracted by proximal inhibition. Am. J. Physiol. Renal. Physiol.; 305:F510-F519. 23720346 10.1152/ajprenal.00183.2013 PMC3891260

[b29] Ponce‐Coria J. Markadieu N. Austin T. M. Flammang L. Rios K. Welling P. A. 2014 A novel Ste20‐related proline/alanine‐rich kinase (SPAK)‐independent pathway involving calcium‐binding protein 39 (Cab39) and serine threonine kinase with no lysine member 4 (WNK4) in the activation of Na‐K‐Cl‐cotransporters. J. Biol. Chem.; 289:17680-17688. 24811174 10.1074/jbc.M113.540518 PMC4067202

[b30] Pu H. X. Cluzeaud F. Goldshleger R. Karlish S. J. D. Farman N. Blostein R. 2001 Functional role and immunocytochemical localization of the γa and γb forms of the Na, K‐ATPase γ subunit. J. Biol. Chem.; 276:20370-20378. 11278761 10.1074/jbc.M010836200

[b31] Pu H. X. Scanzano R. Blostein R. 2002 Distinct regulatory effects of the Na, K‐ATPase γ subunit. J. Biol. Chem.; 277:20270-20276. 11929868 10.1074/jbc.M201009200

[b32] Rengarajan S. Lee D. H. Oh Y. T. Delpire E. Youn J. H. McDonough A. A. 2014 Increasing plasma [K^+^] by intravenous potassium infusion reduces NCC phosphorylation and drives kaliuresis and natriuresis. Am. J. Physiol. Renal. Physiol.; 306:F1059-F1068. 24598799 10.1152/ajprenal.00015.2014 PMC4010681

[b33] Sohara E. Rai T. Yang S. S. Ohta A. Naito S. Chiga M. 2011 Acute insulin stimulation induces phosphorylation of the Na‐Cl cotransporter in cultured distal mpkDCT cells and mouse kidney. PLoS One; 6:e24277 21909387 10.1371/journal.pone.0024277 PMC3164195

[b34] Subramanya A. R. Ellison D. H. 2014 Distal convoluted tubule. Clin. J. Am. Soc. Nephrol.; 910.2215/CJN.05920613 10.2215/CJN.05920613 PMC4255408 24855283

[b35] Sweadner K. J. Gilkeson R. C. 1985 Two isozymes of the Na, K‐ATPase have distinct antigenic determinants. J. Biol. Chem.; 260:9016-9022. 2410405

[b36] Sweadner K. J. Rael E. 2000 The FXYD gene family of small ion transport regulators or channels: cDNA sequence, protein signature sequence, and expression. Genomics; 68:41-56. 10950925 10.1006/geno.2000.6274

[b37] Therien A. G. Karlish S. J. D. Blostein R. 1999 Expression and functional role of the γ subunit of the Na, K‐ATPase in mammalian cells. J. Biol. Chem.; 274:12252-12256. 10212192 10.1074/jbc.274.18.12252

[b38] Tiwari S. Riazi S. Ecelbarger C. A. 2007 Insulin's impact on renal sodium transport and blood pressure in health, obesity, and diabetes. Am. J. Physiol. Renal. Physiol.; 293:F974-F984. 17686957 10.1152/ajprenal.00149.2007

[b39] Vallon V. Schroth J. Lang F. Kuhl D. Uchida S. 2009 Expression and phosphorylation of the Na^+^‐Cl^−^ cotransporter NCC in vivo is regulated by dietary salt, potassium, and SGK1. Am. J. Physiol. Renal. Physiol.; 297:F704-F712. 19570885 10.1152/ajprenal.00030.2009 PMC2739704

[b40] Wang Y. B. Leroy V. Maunsbach A. B. Doucet A. Hasler U. Dizin E. 2014 Sodium transport is modulated by p38 kinase‐dependent cross‐talk between ENaC and Na,K‐ATPase in collecting duct principal cells. J. Am. Soc. Nephrol.; 25:250-259. 24179170 10.1681/ASN.2013040429 PMC3904566

[b41] Wetzel R. K. Sweadner K. J. 2001 Immunocytochemical localization of the Na, K‐ATPase α and γ subunits in the rat kidney. Am. J. Physiol.; 281:F531-F545. 10.1152/ajprenal.2001.281.3.F531 11502602

[b42] Wetzel R. K. Sweadner K. J. 2003 Phospholemman expression in extraglomerular mesangium and afferent arteriole of the juxtaglomerular apparatus. Am. J. Physiol.; 285:F121-F129. 10.1152/ajprenal.00241.2002 12657562

[b43] Wetzel R. K. Pascoa J. L. Arystarkhova E. 2004 Stress‐induced expression of the gamma subunit (FXYD2) modulates Na, K‐ATPase activity and cell growth. J. Biol. Chem.; 279:41750-41757. 15280368 10.1074/jbc.M405622200

